# Regionalized Strategies for Food Loss and Waste Management in Spain under a Life Cycle Thinking Approach

**DOI:** 10.3390/foods9121765

**Published:** 2020-11-28

**Authors:** Daniel Hoehn, Jara Laso, Jorge Cristóbal, Israel Ruiz-Salmón, Isabela Butnar, Aiduan Borrion, Alba Bala, Pere Fullana-i-Palmer, Ian Vázquez-Rowe, Rubén Aldaco, María Margallo

**Affiliations:** 1 Department of Chemical and Biomolecular Engineering, University of Cantabria, Avda, De Los Castros, s.n., 39005 Santander, Spain; daniel.hoehn@unican.es (D.H.); jara.laso@unican.es (J.L.); jorge.cristobal@unican.es (J.C.); israel.ruizsalmon@unican.es (I.R.-S.); maria.margallo@unican.es (M.M.); 2 Institute for Sustainable Resources, University College of London, Central House, 14 Upper Woburn Place, London WC1H 0NN, UK; i.butnar@ucl.ac.uk; 3 Department of Civil, Environmental and Geomatic Engineering (CEGE), University College London, London WC1E 6DE, UK; a.borrion@ucl.ac.uk; 4 UNESCO Chair in Life Cycle and Climate Change, Escola Superior de Comerç International (ESCI-UPF), Pg. Pujades 1, 08003 Barcelona, Spain; alba.bala@esci.upf.edu (A.B.); pere.fullana@esci.upf.edu (P.F.-i-P.); 5 Peruvian Life Cycle Assessment and Industrial Ecology Network (PELCAN), Department of Engineering, Pontificia Universidad Católica del Perú, Av. Universitaria 1801, San Miguel, Lima 15088, Peru; ian.vazquez@pucp.pe

**Keywords:** aerobic composting, anaerobic digestion, food loss and waste, thermal treatment, life cycle assessment, Paris agreement, regionalization

## Abstract

Food loss and waste (FLW) has become a central concern in the social and political debate. Simultaneously, using FLW as a bioenergy source could significantly contribute to closing the carbon cycle by reintroducing energy into the food supply chain. This study aims to identify best strategies for FLW management in each of the 17 regions in Spain, through the application of a Life Cycle Assessment. To this end, an evaluation of the environmental performance over time between 2015 and 2040 of five different FLW management scenarios implemented in a framework of (i) compliance and (ii) non-compliance with the targets of the Paris Agreement was performed. Results revealed savings in the consumption of abiotic resources in those regions in which thermal treatment has a strong presence, although their greenhouse gas (GHG) emissions in a scenario of compliance with climate change targets are higher. In contrast, scenarios that include anaerobic digestion and, to a lesser extent those applying aerobic composting, present lower impacts, including climate change, suggesting improvements of 20–60% in non-compliance and 20–80% in compliance with Paris Agreement targets, compared to the current scenarios.

## 1. Introduction

Renewable energy production policies in Spain are determined by the international context and European Union (EU) recommendations, which are looking for a more sustainable and low-carbon economy to achieve the Paris Agreement targets. Among them, the goal of limiting global warming to well below 2 °C above pre-industrial levels and pursuing efforts to limit it to 1.5 °C stands out [1]. Based on the horizon of the EU being carbon neutral by 2050, the EU has established the specific objectives of reducing greenhouse gas (GHG) emissions by 40% in 2030 as compared to 1990, which includes an aim of having a share of 32% of renewable energy production [2]. Consequently, in 2019, the Spanish government included the EU targets in the draft of the Integrated National Energy and Climate Plan 2021–2030 [3], which aims to integrate the environmental, economic, and social benefits of energy transition in the Spanish economy. To achieve these objectives, the coordination and active involvement of the 17 Spanish regions (i.e., autonomous communities) is essential, considering that Spain has a heavily decentralized legislative system, which implies that decision-making is partially regionalized.

In this framework, the energy produced from non-fossil organic material of biological origin, so called bioenergy, is being promoted as a substitute for non-renewable energy to reduce GHG emissions and dependency on energy imports [4]. Nowadays, bioenergy accounts for ca. 18.5% of renewable energy consumption in the EU [5], but less than 1.1% in Spain [6]. However, from all the sources of bioenergy, including the use of solid biomass, biogas, liquid biofuels, and renewable municipal waste, what kind of resource should be used for power generation is an open question owing to environmental, ethical-social, and economic aspects. Food loss and waste (FLW), which in the present study refers to FLW occurring at every stage of the food supply chain (FSC) [7], has been widely suggested as an alternative to biofuel production [8] due to its organic and nutrient-rich composition, representing a potential global warming mitigation path [9]. In addition, using FLW as a bioenergy source could significantly contribute to a close carbon cycle [10] by reintroducing energy in the FSC [11].

FLW has become a central concern in the social and political debate, as at least one-third of all edible food production is wasted worldwide throughout the entire FSC (20% in the European Union) [12]. This estimate could be higher by a harmonization in the definition of FLW and the collection of FLW generation data [13]. Consequently, the United Nations have adopted the compromise of halving food waste at the retail and consumer level by 2030 and reducing FLW along the FSC, as necessary measures to meet the increasing challenges of feeding the world’s population, raising food security, and achieving environmental sustainability [14,15]. At the EU level, reducing FLW is also a central issue included in the recent European Commission circular economy action plan [16,17]. More recently, the “Farm to Fork Strategy” [18] aims to make food systems fair, healthy and environmentally friendly. In parallel, more than a hundred initiatives to reduce FLW have been implemented in EU countries at national and sub-national levels, through awareness campaigns and training and research programs [19]. Some prominent examples of these programs include “More Food, Less Waste”, in Spain, “Love Food, Hate Waste” from the Waste and Resources Action Program (WRAP) in the UK, and the Milan Protocol promoted by the Foundation Barilla Centre for Food and Nutrition in Italy. However, the potential contribution of FLW to renewable energy generation is often disregarded when discussing FLW management.

Beyond reductions in FLW, the European Commission is promoting the so-called waste hierarchy, positioning prevention at the top [20], and requires Member States to monitor and report on FLW generation and to implement national FLW reduction programs [21]. Additionally, the Directive 1999/31/EC on the landfill of waste asked the EU member states to reduce the share of landfilled biodegradable municipal waste to 75% in 2005, to 50% in 2009 and 35% in 2016 in relation to 1995 [22]. However, there is a high diversity between countries in terms of FLW management strategies. For instance, Denmark, Austria, and Germany are reference countries in terms of avoiding landfilled waste [23]. Nevertheless, while Denmark is focused on strategies of waste thermal treatment (in the present study, refers to incineration with energy recovery) [24], Austria is developing decentralized aerobic composting (AC) systems [25], and Germany is investing in anaerobic digestion (AD) plants for organic waste [26]. In Spain, an important fraction of FLW is still landfilled. The rest is being managed in the 10 existing thermal treatment plants, or in mechanical–biological treatment stations, based on AC or AD systems, whereas pre-treated FLW (i.e., the remaining matter after the treatment) is sent back to landfill or thermal treatment plants. In recent years, the source-separation of the specific AD plants has been reduced to a few pilot projects. Due to the differences in the available technologies and FLW composition, the current study hypothesizes that the FLW management strategies for each Spanish region from an environmental point of view can vary considerably. Moreover, the search for the most optimal strategy within the same region could also be different in a framework of compliance and non-compliance with the Paris Agreement targets. To test these hypotheses, this study aims to determine the potentially most optimal strategies for FLW management in the 17 regions in Spain using prospective Life Cycle Assessments (LCAs). Therefore, the evaluation of the environmental performance over time between 2015 and 2040 of a scenario showing the current FLW management at each region, and five different management scenarios implemented in a framework of (i) compliance (2DS) and (ii) non-compliance with the Paris Agreement targets (BAU), was performed. The study aims to highlight the need of developing regionalized FLW management policies to steer Spanish policymaking to move from a national to a regional approach when developing future roadmaps, as well as integrating FLW management and renewable energy policies.

## 2. Materials and Methods

### 2.1. Goal Definition

This study conducted an LCA following the international standards from the International Organization for Standardization (ISO) 14040 [27] and ISO 14044 [28], to determine the most optimal scenario of FLW management regarding each of the 17 Spanish regions. LCA is a standardized methodology for analyzing the potential environmental impacts of a product, process, or service throughout its life cycle [29], which has been widely applied to improve the design or to optimize a wide range of production processes [30]. The current FLW management in each region is compared to five alternative scenarios regarding the type of FLW treatment, which are described in Section 2.4. The environmental performance of these scenarios was evaluated for the period 2015–2040 considering compliance (2DS) and non-compliance (BAU) with the Paris Agreement targets. The simulations over time are based on the energy mix projections developed by the TIMES Integrated Assessment Model from the University College London (TIAM-UCL). These consider 16 regions covering all the world [31]. For this study, data for the Western European Region, which includes Spain, were used.

### 2.2. Function and Functional Unit

The main function of the system is the management of FLW under different simulated scenarios. In order to measure this function, a suitable functional unit has to be defined, to which all the inputs and outputs are referred. In this case, the treatment of one metric ton of FLW in each Spanish region in the respective year of analysis was assumed as the functional unit.

### 2.3. System Boundaries

**Figure 1:**
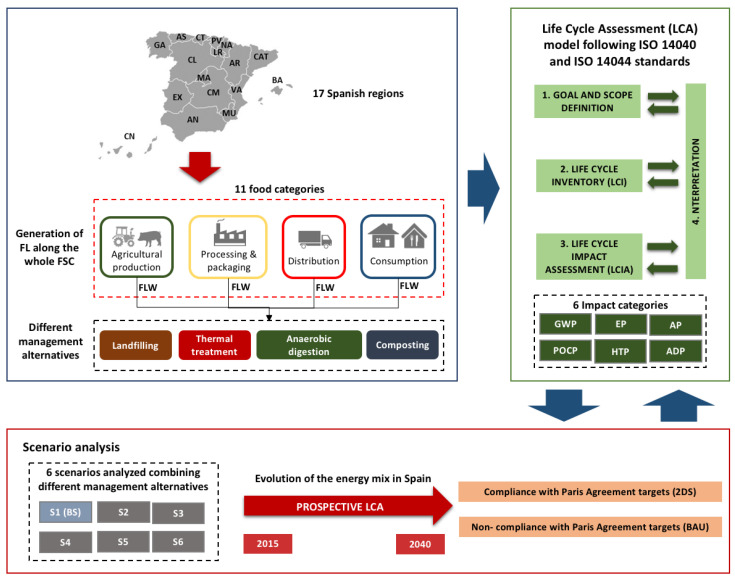
Conceptual diagram of the Life Cycle Assessment methodology developed, based on Aldaco et al. [34]. Regions: AN: Andalusia, AR: Aragon, AS: Principality of Asturias, BA: Balearic Islands, CN: Canary Islands, CT: Cantabria, CM: Castile-La Mancha, CL: Castile and Leon, CAT: Catalonia, EX: Extremadura, GA: Galicia, LR: La Rioja, MA: Community of Madrid, MU: Region of Murcia, NA: Chartered Community of Navarra, PV: Basque Country, VA: Valencian Community, FSC: food supply chain, FLW: food loss and waste, ISO: International Organization for Standardization, GWP: Global Warming Potential, EP: Eutrophication Potential, AP: Acidification Potential, POCP: Photochemical Ozone Creation Potential, HTP: Human Toxicity Potential, ADP: Abiotic Depletion Potential, S1(BS): Scenario 1 (Baseline Scenario), S2: Scenario 2, S3: Scenario 3, S4: Scenario 4, S5: Scenario 5, S6: Scenario 6, 2DS: compliance with the Paris Agreement targets, BAU: non-compliance with the Paris Agreement targets.

This LCA has a cradle to grave approach (Figure 1), including within the system boundaries the FLW generation in the first stages of the FSC—agricultural production, processing and packaging—and FW in the distribution and consumption stages. FLW was divided into 11 categories of food, following the division suggested by the Food and Agriculture Organization of the United Nations (FAO) [32], which considers cereals, sweets, vegetable oils, vegetables, fruits, pulses, roots, dairy products, eggs, fish and seafood, and meat. Collection and transportation of FLW to the different management alternatives were not considered in the system boundaries since it was assumed that all FLW management options had similar environmental loads, due to their low influence. The mass balances from a previous study have been used [33] in order to consider FLW of different food categories. Regarding FLW management, AC, AD, thermal treatment, and landfill were evaluated. To determine the FLW generated in the four stages of the FSC and the amount of FLW treated at each management option, the data published in different Spanish governmental sources have been used. The autonomous cities of Ceuta and Melilla were left out of the scope of the study considering their low demographic weight (<0.4%). Both edible and non-edible FLW fractions collected were also considered.

### 2.4. Scenarios under Study

In order to determine the most optimal FLW management strategies for the 17 regions, six different scenarios, including the baseline scenario (S1), were analyzed within this study by implementing them in all regions and all the analyzed years (as summarized in Table 1).

- Scenario 1 (S1). Represents the baseline scenario taking into account the current FLW management in each region (shown in Table 2), according to data published by the Spanish Waste Management Framework Plan (PEMAR) [35] and the CONAMA Foundation [36]. The results of S1 are calculated using the best-founded data for 2015, combined with certain assumptions.
- Scenario 2 (S2). Replicates the current situation in Germany regarding FLW management [37], where AC represents the high part of the treatment, but AD systems are increasingly being promoted. Therefore, it is considered that 75% of FLW goes to AC, 20% to AD and the rest is divided between landfill (2.5%) and thermal treatment (2.5%).
- Scenario 3 (S3). This scenario prioritizes the use of AD systems, assuming that 75% goes to AD, 20% to AC, and the rest is divided between landfill (2.5%) and thermal treatment (2.5%).
- Scenario 4 (S4). This scenario is based on current Danish conditions, where over 90% of the share of biowaste is incinerated [38]. Thus, 90% of FLW goes to thermal treatment, while the “10%” goes to landfill, AD and, respectively, AC in equal proportions.
- Scenario 5 (S5). This scenario is based on the increasingly promoted claim that FLW is a valuable resource that should never end up in landfilling sites [39]. It is assumed that landfilling is not a FLW management alternative, so 33.3% goes to each of the remaining management options.
- Scenario 6 (S6). Landfilling and thermal treatment are not considered in this scenario, so 50% of FLW is treated in AC, and 50% in AD. The argument for avoid including thermal treatment plants in S6 refers to the fact that, similarly to what has recently occurred to coal plants in many nations including Spain, thermal treatment plants will potentially have problems with regard to providing energy to the system by the year 2030. More specifically, they will have serious difficulties in maintaining competitiveness against other technologies in an environment highly conditioned by the European response to climate change, in which the cost of CO_2_ will tend to be increasingly higher [2].

The scenarios simulated were studied taking into account the evolution of the electricity mix in Spain from 2015 to 2040 in the 2DS and BAU frameworks (as described in Section 2.5).

**Figure 2:**
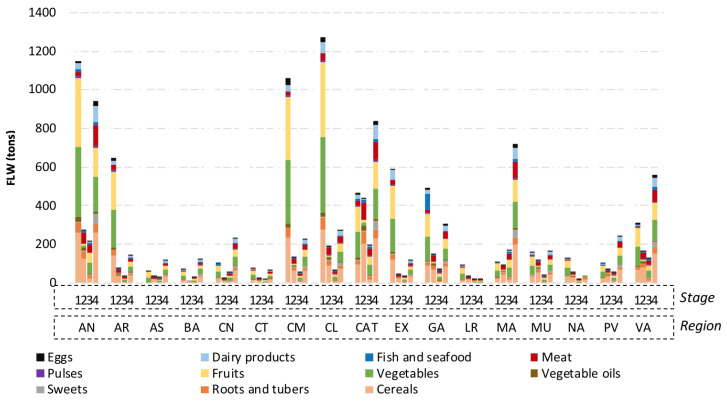
Food loss and waste generation at each region (in tons) in 2015, divided in the 11 food categories and four stages considered. Stages: 1: agricultural production, 2: processing and packaging, 3: distribution, and 4: consumption. Regions: AN: Andalusia, AR: Aragon, AS: Principality of Asturias, BA: Balearic Islands, CN: Canary Islands, CT: Cantabria, CM: Castile-La Mancha, CL: Castile and Leon, CAT: Catalonia, EX: Extremadura, GA: Galicia, LR: La Rioja, MA: Community of Madrid, MU: Region of Murcia, NA: Chartered Community of Navarra, PV: Basque Country, VA: Valencian Community.

For the modelling of FLW generation in each region, the FLW composition was considered (see Figure 2). Therefore, a literature review was conducted in order to determine the management possibilities of each FLW fraction regarding regulatory and technical issues (as shown in Table 3). The highest priority are prevention and re-use, with re-use meaning the use of the materials without further processing—for instance, food donation to charities. AC has regulatory restrictions for animal products and vegetable oils [40]. Therefore, AC was not included for the management of vegetable oils, meat, fish and seafood, dairy products, and eggs. These residues were assumed to go to the main FLW management option in each scenario and, following the waste hierarchy, prioritizing AD and thermal treatment over landfill. Consequently, these fractions were assumed to go to landfilling in S1, to AD in S2, S3, and S6, to thermal treatment in S4, and 50% to thermal treatment and 50% to AD in S5. Moreover, as thermal treatment generates 15–25% of ashes [41], including bottom and fly ashes, an average value of 20% was assumed to go to landfilling in regions with thermal treatment plants.

### 2.5. Life Cycle Inventory

A set of assumptions and calculations were carried out to develop the life cycle inventory (LCI) of FLW generation and management in the 17 regions regarding the four stages of the FSC, the 11 FLW categories, and the four management options considered. To calculate the data for the stages of agriculture production, and processing and packaging, data reported by the Spanish Ministry of Agriculture, Fishery and Food [44,45] were used to determine the percentage of livestock, agricultural or fishery production, as well as the number of existing industries, in each region, from the total values reported. Regarding the distribution and household stages, the calculations were based on the existing population in 2015 [46], adding as a part of the population the number of tourists in each region in that year [47]. Finally, it was assumed that FLW accounts for 49% from total reported waste [36]. A detailed description is reported in Figure 2 and detailed in Table A1 of Appendix A.

The different FLW treatment techniques have been developed according to the models extracted from Aldaco et al. [48]:

- AC was modelled using the professional database of the GaBi software [49], which considers closed halls or so-called composting boxes or rotting tunnels. The input waste is assumed as an average mixture of biodegradable waste consisting of biodegradable garden and park waste, as well as a 35% content of food and kitchen waste. For the selective collection fraction, the composting system includes the energy requirements of a mechanical separation unit [50].
- AD was modelled using the Ecoinvent database [51], including storage of the substrates, anaerobic fermentation, as well as the storage of digestate after fermentation. One cubic meter of biogas is assumed to produce 2.07 kWh of electricity [52].
- Thermal treatment was based on the professional database of the GaBi software [49] for the biodegradable waste fraction of municipal solid waste. To model a single fraction, energy production and credits were attributed to the biodegradable waste fraction. The plant consists of an incineration line fitted with a grate and a steam generator. Grate is the most common technology in Europe, applied in 80% of plants in Spain [53]. The thermal treatment of one metric ton of waste produces 495 MJ of energy, 1277 MJ of steam, 220 kg of bottom ash, and 42 kg of boiler ash, filter cake, and slurries.
- Landfill with biogas recovery includes biogas and leachate treatment and deposition. Sealing materials (e.g., clay or mineral coating) and diesel for the compactor were also included. The modelling was based on the landfill process for municipal household waste from the professional database of GaBi [49]. According to the model, 17% of the biogas naturally released is collected, treated, and burnt to produce electricity. The remaining biogas is flared (21%) and released to the atmosphere (62%). A rate of 50% transpiration/runoff and a 100-year lifetime for the landfill were considered. Additionally, a net electricity generation of 0.0942 MJ per kg of municipal solid FLW was assumed [49].

**Figure 3:**
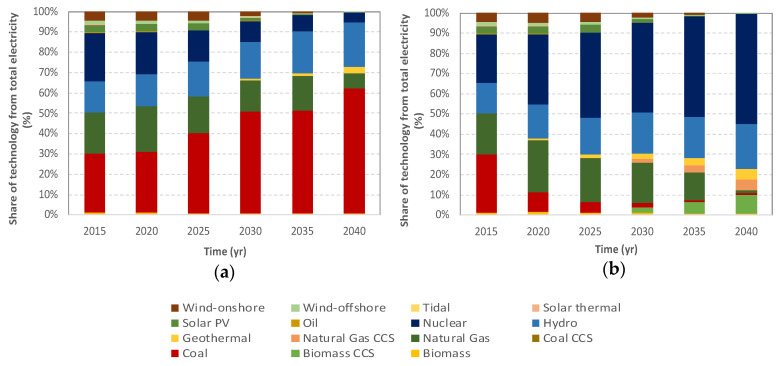
Energy mix simulations according to the TIAM-UCL model for Western European region. (**a**) Simulated BAU and (**b**) 2DS energy mix frameworks from 2015 until 2040. PV: photovoltaic, CCS: carbon capture sequestration. Biomass includes waste-to-energy technology such as thermal treatment.

The avoided burden for electricity from AD, thermal treatment, and landfill, are based on the electricity mix simulations according to the TIAM-UCL model, which are shown in Figure 3. The evolution in a BAU framework suggests continuous increase in the energy produced from coal, reaching around 60% of the total energy generation by 2040, followed by hydropower (20%), and natural gas, with less than 10% (as seen in Figure 3a). Biomass and biomass with carbon capture sequestration will begin to decrease starting in 2025 until they almost disappear by 2040. Regarding the evolution in a 2DS framework (Figure 3b), surprisingly, nuclear power seems to have an enormous increase, reaching 55% of the total electricity mix in 2040, followed by hydropower (20%) and onshore wind (10%). This highlights that certain decarbonization policies in the electricity sector may foster the rise of a controversial energy source (i.e., nuclear), which opens the debate on whether the final outcome justifies any strategy to meet the Paris Agreement targets. This fact suggested another piece of policy advice, which would be complementary and necessary together with climate policies, concerning existing previous experiences such as the ban of nuclear power developed in 1978 in Austria [54]. Finally, both options suggested a reduction in the energy generated by biomass in 2025, which nearly disappears by 2040.

### 2.6. System Expansion

The scenarios under study are multi-output processes in which the management of FLW is the main function of the system and the production of electricity, steam, and compost are additional functions. Therefore, the environmental burdens must be allocated among the different functions. To handle this problem, ISO 14040 establishes a specific allocation procedure in which system expansion should be prioritized [27]. The energy produced in waste decomposition (i.e., landfill and AD) and combustion (i.e., thermal treatment) was assumed to substitute the equivalent amount of electricity from the grid. The electricity recovered in all scenarios was assumed to be sent to the national grid, displacing electricity from the average electricity mix. However, this value could be lower if energy losses and uses for other purposes are considered. Moreover, the environmental credits of compost are also considered. Compost is assumed to replace mineral fertilizer, with a substitution ratio of 20 kg N equivalent per metric ton of compost [55]. The fertilizer production as total N was obtained from the professional database of the GaBi software [49].

### 2.7. Life Cycle Impact Assessment

In order to quantify the potential environmental impacts of the scenarios modelled, six environmental impact categories (shown in Table 4) were selected from the v.306 methodology of the Institute of Environmental Sciences of Leiden University, in the Netherlands [56]. This choice was made considering that the assessment method has enough scientific endorsement and is widely used in the LCA literature [57].

The selection of impact categories was carried out considering the most relevant impacts linked to organic waste and its treatment. In this sense, climate change, due to anaerobic organic decomposition, was highlighted as an important indicator to be considered. The presence of different waste treatment technologies, namely thermal treatment, pushed towards the inclusion of human toxicity and air quality categories, such as photochemical oxidation. Acidification and eutrophication were selected due to the presence of acidic gases and high amounts of nutrients in FLW, respectively. Finally, abiotic depletion was modelled considering the displacement of fossil fuels and resources in the systems in which electricity and fertilizers are generated from FLW. It is acknowledged that other assessment methods could have been chosen to conduct certain impact categories, but the use of one single method constructed with the same methodological basis was prioritized.

### 2.8. Main Limitations and Assumptions of the Study

The main limitation of the present study is the uncertainty in the data used, the main sources of uncertainty being the amounts of FLW generated and the type of management for the different FLW categories in the reference year, as well as the trends until and during the modelled time. Moreover, it is difficult to link FLW generation and management, as the whole process takes time and in the meantime a fraction of the mass might be lost (e.g., due to drying). Differences can also occur due to import and export of waste, as well as unaccounted fractions. Moreover, although information is available regarding the different treatment and disposal methods, existing statistics generally refer to the generation of biodegradable municipal waste, not to the generation of biowaste or FLW [55]. Biodegradable municipal waste also includes paper, cardboard, and biodegradable textiles. Additionally, in the more advanced stages of the FSC, FLW is usually mixed with general waste, which complicates the determination of the percentage that corresponds to FLW exclusively. In this framework, the modelling of the incineration process of FLW has a considerable degree of uncertainty, as the provided processes are not specifically adapted to individual waste streams, and biodegradable waste was used instead of FLW, which means a partially different heating value. The combustion of FLW produces dioxins and furans depending on ranges of temperatures (from 250 to 400 °C). Nevertheless, one limitation of toxicity categories in LCA is that most of the methods do not include a characterization factor for these pollutants, providing an uncertainty source in the results. This limitation was found in the CML method, but it is common in other impact methods, such as Recipe—both of them being widely applied for LCA practitioners. Moreover, is important to highlight that the assumed source-separation of the FLW mentioned fractions (described in Section 2.6) is a mainly theoretical process, with the exception of some industrial waste streams. Additionally, how the difference of FLW composition will affect its management has only been considered regarding the restrictions in the use of animal products in AC. Other factors such as biogas generation and moisture content have not been included in the calculations. How these aspects would influence the management process would be another relevant element to include within the system boundaries, which was not analyzed in this work. The amount of FLW also depends on factors such as the time of the year and the region. Thereby, this study deals with a field where there are important gaps in the clarity of the reported data, both in terms of the generated quantities of FLW, and in terms of the relative importance of different recovery or disposal options. The AC process considers the use of the digestate in soils, thereby avoiding the use of fertilizers. Nevertheless, the potential methane emissions due to the direct use in soil have not been assessed. In addition, it is important to highlight that the positive impact on the environment provided by compost is underestimated by current LCA methodology when it is compared to digestate. This is due to the fact that when digestate after AD processing is employed, most of the carbon content is already used as methane and the quality of digestate cannot be compared to compost.

A debatable assumption made in this study concerns the selection of the LCA approach to solve the multi-functionality issue mentioned in Section 2.6. This study has used an attributional approach in which the electricity produced within the system boundaries is sent to the grid, and thus the system is credited with the impacts of producing that amount of electricity using average data from the electricity mix. On the other hand, the selection of a consequential approach would have identified the marginal technology from the mix displaced by the energy produced within the system boundaries, and thus the system would be credited with the impacts of producing that amount of electricity using the displaced technology. According to the literature, the selection of one approach or the other can have an important effect on results and conclusions drawn from LCAs for solid waste management systems [58]. Moreover, technological developments related to the FLW management methods, such as improving the electricity production efficiency or cleaning exhaust gas technology, were not considered in this analysis.

Additionally, the evolution of FLW generation until 2040 was first considered using a logarithmic regression based on the projection of the World Bank Group [59] regarding the Spanish population growth. Thereby, a progressive and cumulative increase was assumed, reaching 6.7% in 2040 compared to 2015. Given the construction of the scenario simulation model, this increase did not generate any change; hence, this process was omitted from the methodology. For the same reason, the Sustainable Development Goal 12.3 target, aiming to reduce food waste by 50% by 2030, and which was an important reason for recent EU legislation which set an obligation for EU member states to measure and report food waste along the FSC from 2020 onwards [60], was not included in the modelling process. Both facts may be another source of uncertainty and limitation in the results of this work.

Finally, it is important to remark that each simulation will always represent a simplification of reality.

## 3. Results and Discussion

Within this section, results from two different analyses are presented and discussed. Section 3.1 is focused on the current Spanish regional FLW management configuration (scenario 1). The environmental performance of the 17 regions is analyzed considering future periods and maintaining the configuration of scenario 1 under different political decisions (i.e., fulfill the Paris Agreement targets or not). Section 3.2 is focused on the possibility of changing the FLW management configuration (scenarios 2 to 6) also under different political decisions (i.e., fulfill the Paris Agreement targets or not), analyzing the environmental performance and a regionalized analysis of the GHG impact category, as an example, for those configurations. Finally, Section 3.3 presents a comparison of the results with previous published studies within this topic.

### 3.1. Environmental Impacts of the Current Spanish Regional FLW Management: Scenario 1

**Figure 4:**
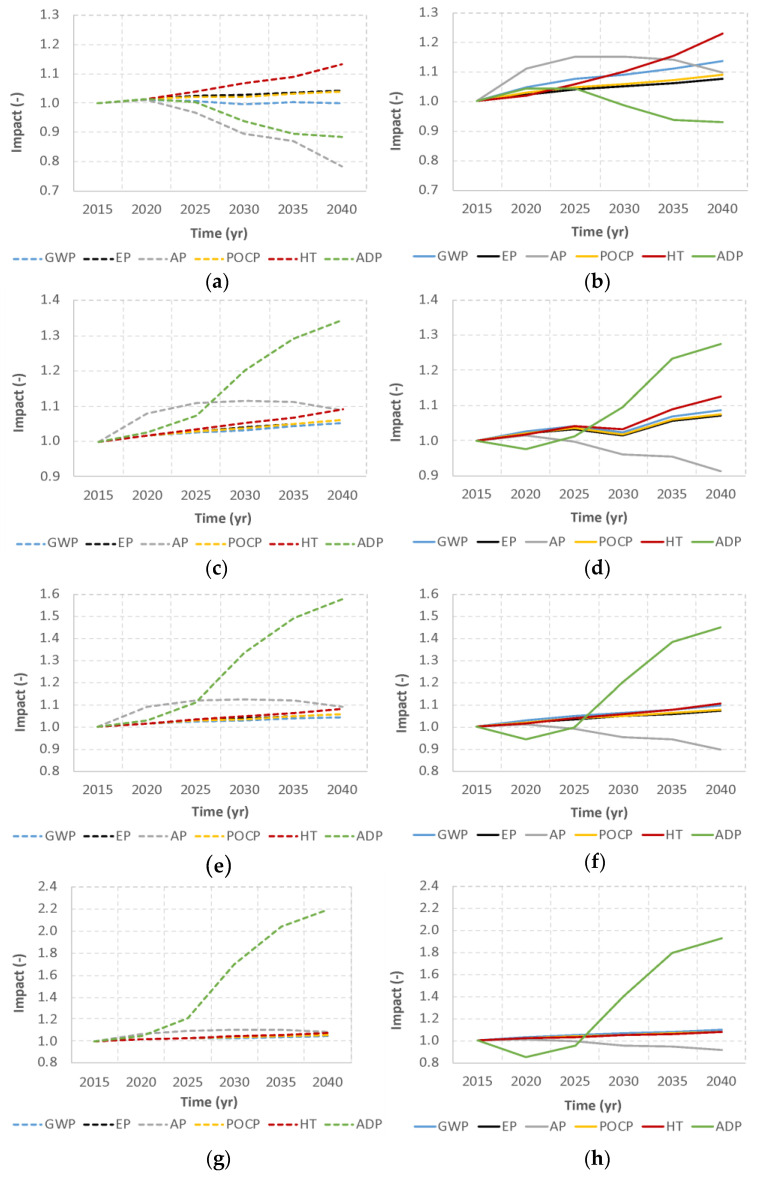
Environmental impacts of current FLW management grouped around main FLW treatment in four clusters. All impacts are normalized by their values in 2015. CT region, as representative for high incineration: (**a**) BAU, (**b**) 2DS; EX region, representative for landfilling with energy recovery: (**c**) BAU, (**d**) 2DS; VA region, representative for a mix of landfilling and AD: (**e**) BAU, (**f**) 2DS; NA region, representative for AC: (**g**) BAU, (**h**) 2DS. GWP: Global Warming Potential (excluding biogenic carbon), ADP: Abiotic Depletion Potential, EP: Eutrophication Potential, HTP: Human Toxicity Potential, POCP: Photochemical Ozone Creation Potential.

Due to the heterogeneity of the management strategies implemented in the 17 Spanish regions (shown in Table 2), which would be maintained until 2040 for this scenario, the environmental performance results differ greatly between regions. In order to see if the future environmental performance is better or worse, impact results by category for a future time period are represented as the ratio between the impact for that period and the impact in 2015. Herein, results are discussed according to two different variables: the influence of the FLW management technologies and the influence of the Paris Agreement framework (reflected in the evolution of the electricity mix). Attending the trends and similarity in results, regions are clustered. Results for one representative region are depicted in Figure 4.

**Figure A1:**
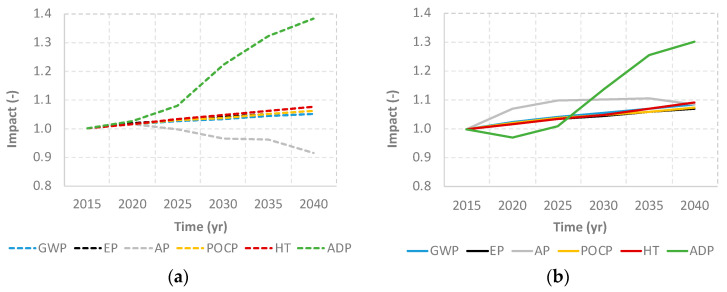

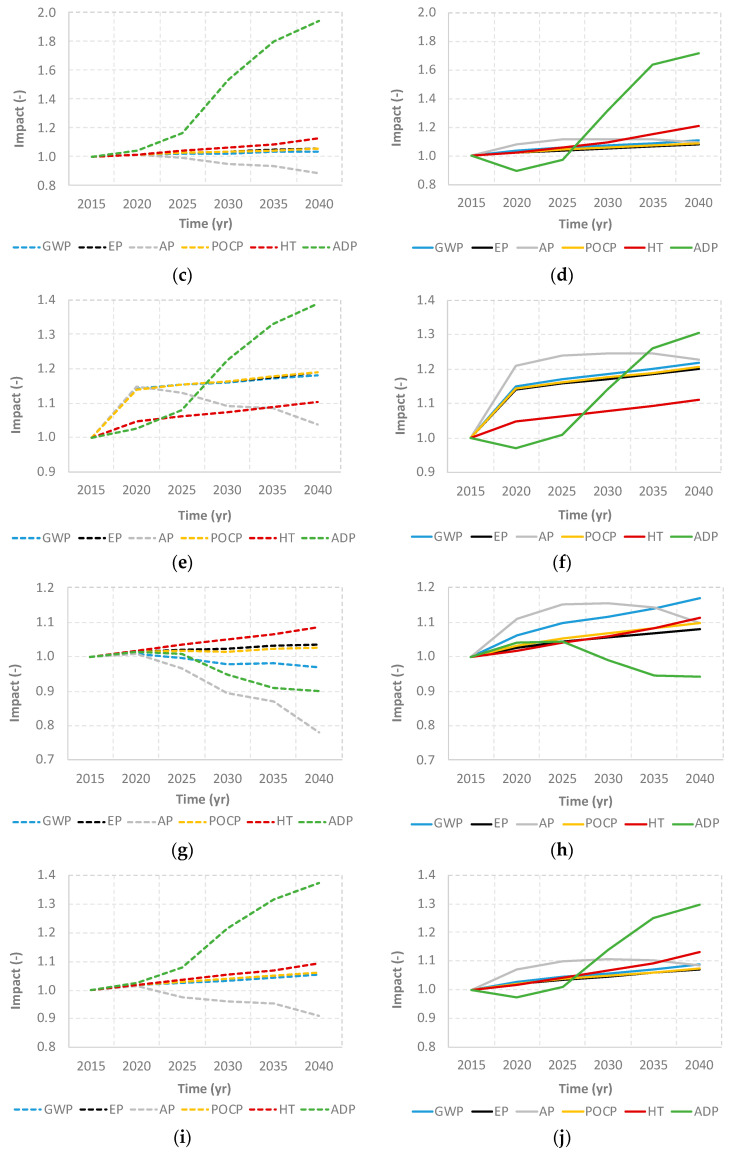

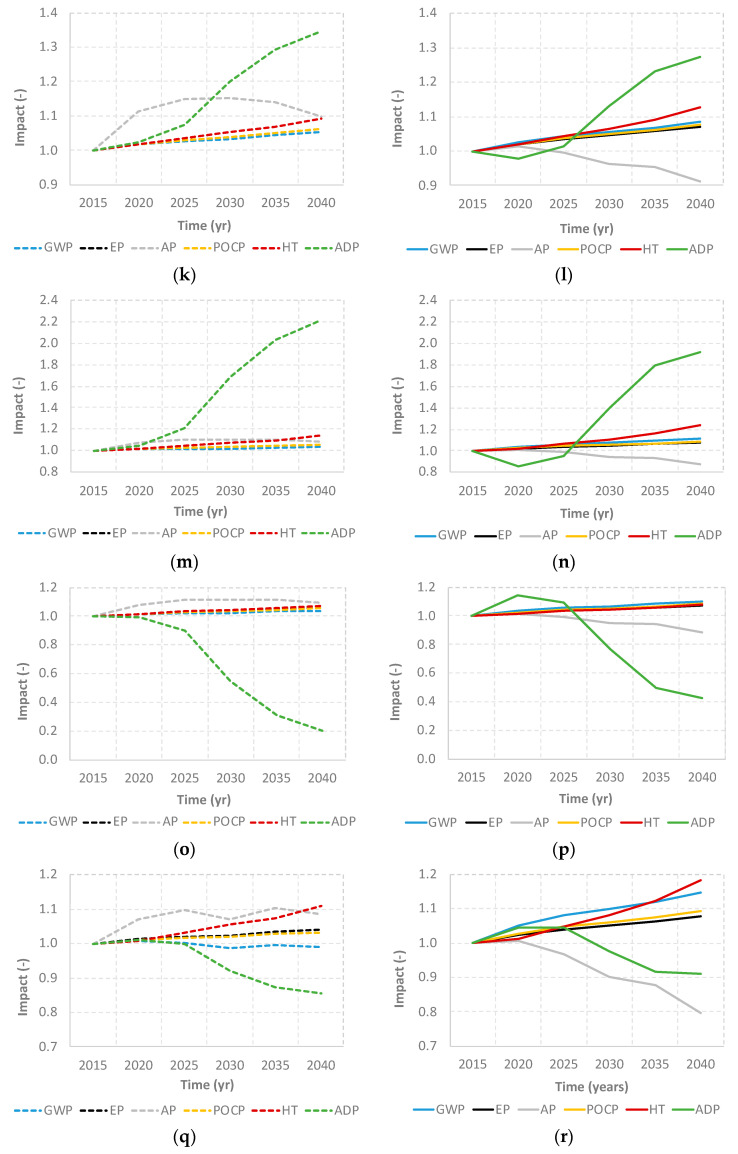

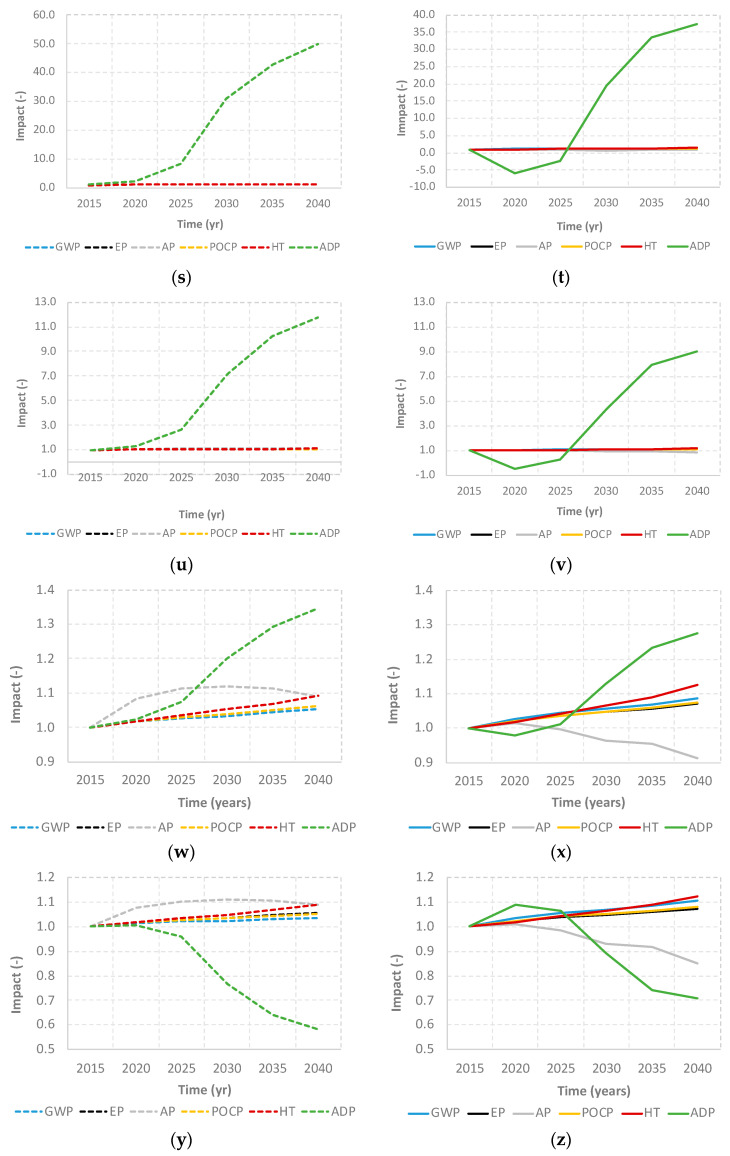
Environmental impacts of current FLW management. AN: (**a**) BAU, (**b**) 2DS; AR: (**c**) BAU, (**d**) 2DS; AS: (**e**) BAU, (**f**) 2DS; BA: (**g**) BAU, (**h**) 2DS; CN: (**i**) BAU, (**j**) 2DS; CM: (**k**) BAU, (**l**) 2DS; CL: (**m**) BAU, (**n**) 2DS; CAT: (**o**) BAU, (**p**) 2DS; GA: (**q**) BAU, (**r**) 2DS; LR: (**s**) BAU, (**t**) 2DS; MA: (**u**) BAU, (**v**) 2DS; MU: (**w**) BAU, (**x**) 2DS; PV: (**y**) BAU, (**z**) 2DS.

In order to see the influence of the different FLW management technologies, one cluster of regions can be made for Cantabria (CT), Balearic Islands (BA), Galicia (GA), and Basque Country (PV), where thermal treatment plays an important role (more than 25%). Thus, results show that the use of thermal treatment is related to a significant decrease in Abiotic Depletion Potential (ADP) (up to 15%), as it presents the highest level of energy generation and, therefore, the greatest savings in terms of resource consumption are achieved. Figure 4a,b show the results for CT under BAU and, respectively, 2DS, whereas BA, GA, and PV, with a similar trend, are included in Figure A1 of Appendix B.

A second cluster of regions can be made for Andalusia (AN), Principality of Asturias (AS), Castile-La Mancha (CM), Canary Islands (CN), Extremadura (EX), and Region of Murcia (MU), where FLW management is carried out almost entirely through landfilling with energy recovery (between 92% and 100%). Figure 4c,d show the environmental burdens for EX under BAU and, respectively, 2DS; the rest of regions are represented in Figure A1 of Appendix B. Results show that the use of landfilling is related to a significant increase in ADP (up to 35%) under both BAU and 2DS futures.

A third group of regions are those that combine the use of landfilling and AD, i.e., Aragon (AR), Castile and Leon (CL), La Rioja (LR), Community of Madrid (MA), and Valencian Community (VA), with AD percentages ranging from 22% to 67%. All these obtained results are similar, with an increase in the consumption of ADP over time (up to 58% for VA, as shown in Figure 4e). The higher the percentage of AD, the higher the ADP values (Figure A1 of Appendix B).

A fourth group of regions is composed of Catalonia (CAT) and Chartered Community of Navarra (NA), in which AC reaches values of 19% and 12%, respectively (Figure 4g for NA). This figure suggests the biggest increase in ADP, higher than that observed in the previous clusters, although the trends are similar to clusters 2 and 3. This is related to the fact that it is the only management option that does not generate energy, and therefore the consumption of abiotic resources through the energy mixes increases much more than in the previous clusters. According to the developed model, generating organic fertilizer through AC, which constitutes an avoided burden with respect to the environmental impacts of the fertilizer that would normally be used, has a much lower importance, in terms of ADP, than not generating energy that would displace other non-renewable sources in the energy mix.

Concerning the impact categories of Global Warming Potential (GWP), Eutrophication Potential (EP), Photochemical Ozone Creation Potential (POCP) and Human Toxicity (HT), there is always a slight increase over time across all clusters. This increase is more pronounced in FLW management configurations that present high rates of incineration.

Analyzing the influence of the Paris Agreement framework on the results, the relation is clear for some impact categories and technologies. The energy mix has a great influence on the Acidification Potential (AP) impact category. Results show a big decrease in AP for the 2DS when landfill is the main technology in the FLW configuration (clusters 2 and 3), since the electricity mix, with higher weight of renewable sources, has a lower environmental burden in AP. However, this is not visible in the configuration with a high share of incineration (cluster 1). In this case, Figure 4a shows a reduction in AP in the BAU scenario (up to 21% in the case of GA), in which the energy from FLW thermal treatment has a lower load of acid gases than the one that would be obtained from the energy mix strongly marked by the presence of fossil fuels such as coal and natural gas. Under 2DS, the reduction in acid gases related to the electricity mix is overcompensated by the acid gases from incinerating FLW, resulting in increased AP.

Regarding ADP, values are always positive (increase from 2015) and higher for BAU comparing to 2DS, except for the configurations in which thermal treatment plays a key role. In the latter case, Figure 4a,b suggest a reduction in the consumption of abiotic resources, which is less pronounced in the 2DS framework (up to 9%), since the energy obtained from thermal treatment is replaced by cleaner energy that uses more renewable sources. This entails lower environmental savings or avoided burdens and, thus, a higher impact is obtained. This is the case for CT, BA and GA and PV, which reduced up to 15% of ADP impact in the BAU framework.

Concerning the impact categories of GWP, EP, POCP and HT, as mentioned before, there is always a slight increase over time, and values are higher under compliance of the Paris Agreement targets (i.e., 2DS) comparing to BAU. The main reason is that the avoided burdens of cleaner energy according to the energy mix of the 2DS framework report lower credits comparing to BAU (see, e.g., Figure 4g,h for NA). These impacts presented higher values (up to 11%, 9%, 23%, and 9%, respectively) in the 2DS scenario, considering that the energy produced from FLW thermal treatment had higher burdens than the cleaner energy that it replaces as avoided charges. Thus, for this management scenario, the compliance with the Paris Agreement targets would penalize thermal treatment. Conversely, thermal treatment implementation would be reinforced in an undesired scenario of progressive increase in emissions of CO_2_ associated with the energy mix until the year 2040. In comparative terms, only the regions with the presence of FLW thermal treatment show a reduction in the consumption of abiotic resources (ADP) related to the ones in which such technology is not present. This is due to the higher energy efficiency of thermal treatment and, therefore, the resources avoided in obtaining energy according to the energy mix projections. Furthermore, this is even more evident in the BAU framework, with a higher consumption of non-renewable resources. The remaining impacts are higher when the thermal treatment is included within the FLW management alternatives, showing the lowest environmental burdens in the regions where AD and AC are used. This is especially remarkable when complying with the Paris Agreement targets, in which the impact is only reduced in regions without thermal treatment plants, since FLW combustion emits a higher amount of acid gases and particles than to obtain their equivalent energy considering the mix in the 2DS scenario.

### 3.2. Alternative Simulated Scenarios Analysis

**Figure 5:**
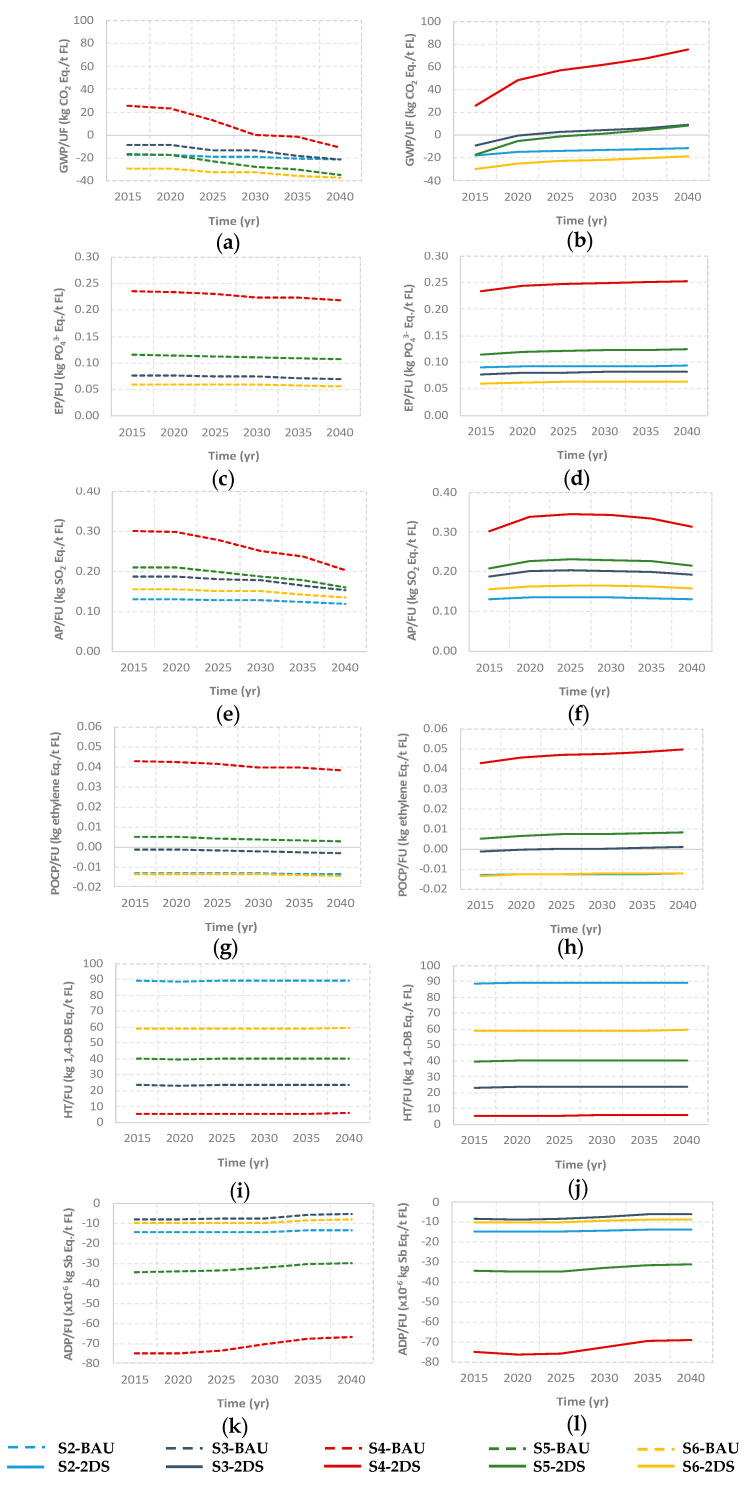
Life Cycle Impact Assessment for the considered FLW management scenarios. Global Warming Potential (GWP): (**a**,**b**); Eutrophication Potential (EP): (**c**,**d**); Acidification Potential (AP): (**e**,**f**); Photochemical Ozone Creation Potential (POCP) (**g**,**h**); Human Toxicity (HT): (**i**,**j**); Abiotic Depletion Potential (ADP): (**k**,**l**). Figures on the left represent BAU, and on the right 2DS.

This section analyzes the environmental performance of changing the FLW management configuration through reducing landfilling and increasing the other technologies as introduced in Section 2.4. First, the different configurations are analyzed per ton of FLW managed. Figure 5 presents the results obtained for GWP, AP, EP, POCP, HT, and ADP for scenarios 2–6 in the BAU and 2DS approaches (measured in kilograms of reference substance per ton of FLW).

In line with the results of scenario 1 discussed in Section 3.1, thermal treatment of organic matter, as an alternative to landfill, represents the scenario with the highest environmental burdens in terms of GWP, EP, AP and POCP, both in the BAU (Figure 5a,c,e,g,i,k) and 2DS (Figure 5b,d,f,h,j,l) frameworks (a comparison of S1 with the rest will be shown in Section 3.3). It acquires special significance if the Paris Agreement targets are achieved, where the energy recovered results in GHG emission rates that could be three times higher until 2040 due to the displacement of clean energy. Scenario S5 (in green), which diversifies FLW treatment strategies between thermal treatment, AC, and AD, is an alternative that, from a comprehensive FLW management perspective (including the inorganic fraction), is attractive. This scenario is strongly influenced by the emissions associated with thermal treatment, being less attractive if the management of the organic fraction is addressed alone. Concerning HT and ADP, both are negatively influenced by the presence of AC and AD in the FLW management option, respectively. This shows the existing trade-off between the different impact categories to be considered by decision-makers.

**Figure 6:**
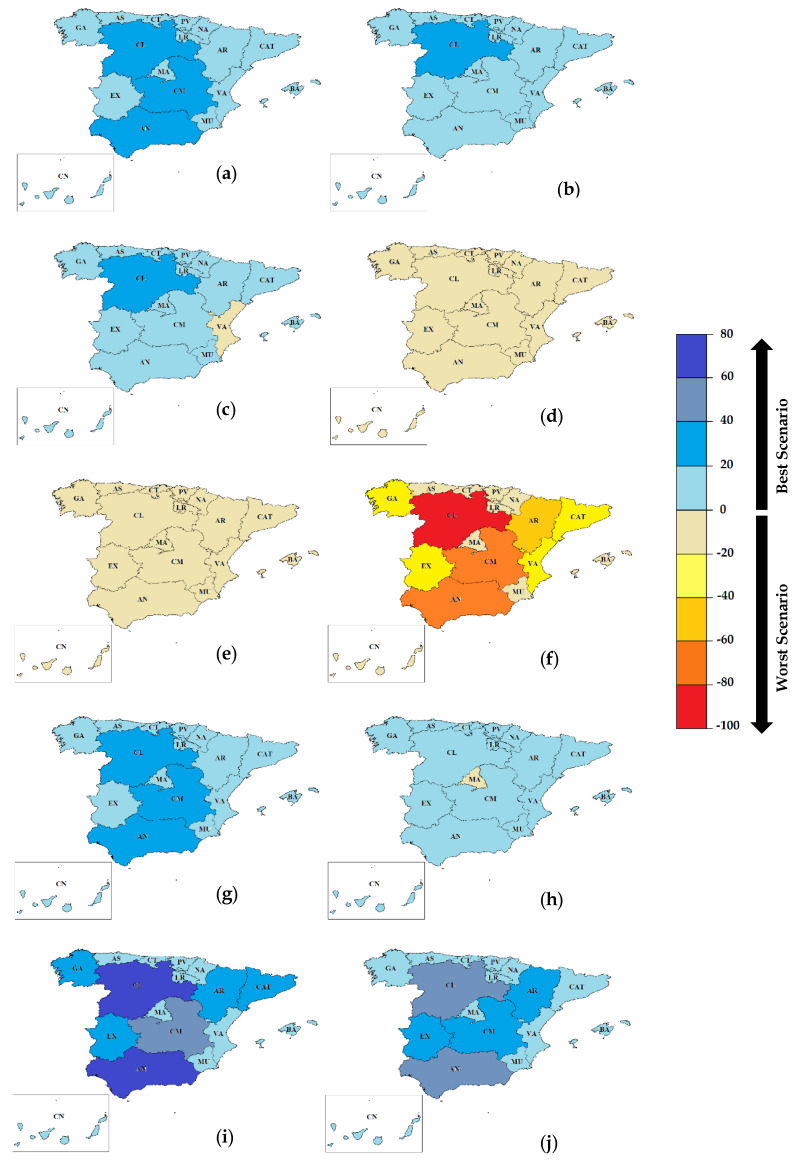
Relative variation (%) of greenhouse gas (GHG) emissions as compared to the current scenario (S1) per region for the considered FLW management scenarios. Scenario S2: (**a**) BAU and (**b**) 2DS; scenario S3: (**c**) BAU and (**d**) 2DS; scenario S4: (**e**) BAU and (**f**) 2DS; scenario S5: (**g**) BAU and (**h**) 2DS; scenario S6: (**i**) BAU and (**j**) 2DS.

In the same line of the analysis performed in Section 3.1, Figure 6 shows the regional evolution of the environmental performance in 2040 for the new FLW configurations (scenarios 2–6) in terms of GWP represented as the variation of percentages between the impact in 2040 and the impact in 2015. Both BAU and 2DS frameworks are analyzed (Appendix B contains the results for the rest of the impact categories).

The results show how the alternative for energy recovery (i.e., S4, thermal treatment share 90%) worsens the GWP by up to 20% in all regions in the BAU framework. If compliance with the Paris Agreement targets is attained (i.e., 2DS), a worsening in GWP is also general for all regions, but in this case the energy recovery from FLW implies an increase in GWP of approximately 60% for AN and CM, in which the management strategy would go from landfilling to thermal treatment, and higher than 80% for CL, in which thermal treatment would replace landfilling and AD. However, the latter case can only be approached as a theoretical reference, as in the other regions in which there are already management options other than landfilling, and in which its replacement in the short or medium term has no practical value.

Discarding the substitution of landfill by thermal treatment, all the other scenarios present significant improvements compared to the current scenarios, reaching improvements through AD and AC (i.e., S6) above 60% for CL and AN in the BAU framework (above 80% in compliance with the Paris Agreement targets), higher than 40% for CM under the BAU framework (above 60% in compliance with the Paris Agreement targets), and around 20% in practically all the other regions. The analysis of the results for the rest of the impacts studied showed a similar trend, as shown in Table A2, Table A3, Table A4, Table A5 and Table A6 in Appendix A.

Consequently, decisions on investment in technologies in the future need to be regional instead of national and always attend to environmental and technical criteria (such as those presented in this work) and oversimplistic and short-term political evaluations. This could be an important path for future research on regional planning, considering other factors as the transport costs, the spatial occurrence of specific FLW generators (such as primary production or food processing and packaging industry), the regional demand (e.g., for energy, for compost), the acceptance of society (e.g., related to source-separation), the on-site demand for energy not connected to season, as well as the physical and chemical characteristics of FLW.

### 3.3. Comparison with the Literature

AD coupled to AC, which was revealed in this study as the path with the highest reduction across all analyzed impacts (i.e., S6), has also been highlighted as an efficient alternative technology, combining biofuel production (i.e., biogas from AD) with sustainable waste management [61,62], as long as the produced biogas is utilized for energy substitution [63]. Different comparative studies, analyzing landfilling, thermal treatment, and AD scenarios, showed similar conclusions, highlighting AD (i.e., S3) as the most favorable alternative in terms of GWP [64] and Energy Return on Investment—EROI [65]. Moreover, a study conducted in Sweden [66] suggested that AD, with the use of biogas and digestate as substitution for vehicle fuel and chemical fertilizers, respectively, resulted in higher avoidance of GWP and POCP, compared to AC or thermal treatment of FLW. Regarding the comparability between AD and AC, the current LCA methodology underestimates the positive impact on environment provided by compost (e.g., there is no accounting of the improved water holding capacity, improved pore volume, increased biodiversity of soil organisms or higher content of stable organic matter through use of compost). In fact, when digestate after AD processing is used, most of the carbon content is already used as methane and the quality of digestate cannot be compared to compost. Therefore, it could be assumed that the positive impact of compost is undervalued in general, in comparison to digestate coming from AD. Thus, the environmental benefits from AD may have shown higher values. An Arcadis report [55] stated that a switch from landfill and thermal treatment is favorable for both AC and AD. Moreover, it also showed that from an economic point of view in terms of treatment costs, switches to AC are more advantageous than to AD, outweighing the fact that the environmental benefits are generally higher for AD. The AC option alone (i.e., S2), has also been presented in the literature as an environmentally friendly and sustainable alternative to manage organic solid wastes [67]. 

Although, in general, thermal treatment (i.e., S4) has gained a bad reputation due to certain environmental impacts, such as the emissions of acid gases, dioxins and furans (PCDD/F), as well as GHG emissions [68], there are other comparative studies [38,69] that suggest lower environmental impacts related to thermal treatment as compared to AD coupled with AC. Regarding the diversity of conclusions for apparently similar scenarios, a review including 25 comparative LCA studies addressing FLW treated in landfills, thermal treatment plants, AC (small and large scale), and AD [70], suggested that the GWP results vary largely amongst the studies. These differences could be due to the definition of the system boundaries and methodological choices or the variations in the input data, as they may not only analyze the category of organic waste, but also fractions of higher calorific waste for production of solid recovered fuels, with higher energy generation rates through thermal treatment.

Finally, the results of the current study reinforce the general consensus in the literature by highlighting that landfilling scenarios, with and without energy recovery, are those that present the highest environmental impacts [71]. Hence, regions that still orient their waste management policy towards landfilling are those with the highest potential for the development of novel waste management policies calling for a reduction in the quantity of biodegradable waste landfilled [72].

## 4. Conclusions

The management of FLW in Spain is highly regionalized, and presents as many scenarios as regions and treatment models associated. In this context, it is not possible to define from a technological and environmental point of view a single common centralized strategy for the entire management of FLW in Spain beyond establishing harmonized guidelines and criteria that facilitate both the transition to a circular economy and the reduction in environmental impacts, especially those associated with global warming.

Results highlighted how the alternative for energy recovery worsens the GWP in all regions in the BAU and 2DS frameworks by up to 20% and between 60% and 80%, respectively. All the other scenarios presented significant improvements (20–60% in BAU and 20–80% in 2DS frameworks) compared to the current scenarios. Thus, the regionalization of FLW management strategies is corroborated in this study as a way forward in upcoming decades, which should be transcribed in an increasingly regional decision-making capacity for policy-makers, focusing firstly on regional criteria and characteristics of the FLW management systems, instead of national plans seeking uniformity of strategies.

Despite the importance of achieving compliance with the EU landfill reduction targets, in general terms, landfilling with energy recovery is the most used technology in Spain with an average of 71%, with some regions reaching up to 100% of FLW management. Promoting this technology, however, both in a 2DS and BAU framework, would increase the environmental impacts in the short and medium term, including GHG emissions by 15%, while the consumption of resources would increase significantly, not complying with the principles of a circular economy. Only those regions in which thermal treatment has a strong presence showed savings in the consumption of resources, although their contribution to global warming under 2DS is higher, as the energy obtained in thermal treatment is not as clean as the one it replaces based on the consumption of non-fossil resources. The results obtained from the scenarios simulated concluded that, on average, those scenarios that include AD and to a lesser extent AC have the lowest impacts—even regarding GHG emissions. Therefore, they comply with the principles of the circular economy and are, also, the most sustainable option from an environmental point of view. In this general context, it is necessary to promote strategies conducive to the source-separated and selective collection of FLW.

Nevertheless, for developing decision-making processes for each region, not only an environmental assessment but also a socioeconomic evaluation is needed. These complementary studies would help guarantee the competitiveness of novel strategies, which could be driven by new financial support derived from sources such as the European Commission’s recently presented Farm to Fork Strategy or the future CAP 2021–2027. For instance, certain variables, such as previous and future investment in waste infrastructure, maintenance of the installations, and transport distances of FLW, may be decisive when thinking of developing or not developing potential new strategies of FLW management.

Overall, the results of this study reinforced the increasingly promoted claim that FLW is a valuable resource that should not end up in landfills, although prevention and valorization should be prioritized over any other management option, in order to move towards a circular economy in the food sector and, thus, contribute to the mitigation of climate change and other environmental impacts.

## Figures and Tables

**Table 1:** Simulated scenarios of food loss (FLW) management in Spanish regions. Scenarios S2 to S6 comply with the Directive 1999/31/EC on the landfill of waste [22]. AD: anaerobic digestion, AC: aerobic composting. Empty spaces mean zero percent

Scenarios	Landfill	Thermal Treatment	AD	AC
S1	Dependent on each region (see Table 2)			
S2	2.5%	2.5%	20%	75%
S3	2.5%	2.5%	75%	20%
S4	3.3%	90%	3.3%	3.3%
S5	-	33.3%	33.3%	33.3%
S6	-	-	50%	50%

**Table 2:** Amount of food loss (FLW) by treatment and region in 2015. Data represented in percentages calculated from mass balances in metric tons reported for each region. Regions: AN: Andalusia, AR: Aragon, AS: Principality of Asturias, BA: Balearic Islands, CN: Canary Islands, CT: Cantabria, CM: Castile-La Mancha, CL: Castile and Leon, CAT: Catalonia, EX: Extremadura, GA: Galicia, LR: La Rioja, MA: Community of Madrid, MU: Region of Murcia, NA: Chartered Community of Navarra, PV: Basque Country, VA: Valencian Community, and SP: Spain. Empty spaces mean zero percent.

Region	Landfill	Thermal Treatment	AD	AC
AN	93.8%	-	2.3%	3.9%
AR	62.0%	-	3.0%	-
AS	92.1%	-	-	7.9%
BA	18.9%	72.7%	5.1%	3.3%
CN	95.7%	-	4.3%	-
CT	35.1%	64.9%	-	-
CM	100%	-	-	-
CL	56.4%	-	43.6%	-
CAT	49.4%	18.4%	15.7%	16.5%
EX	100%	-	-	-
GA	33.6%	50.6%	14.9%	0.9%
LR	35.1%	-	64.9%	-
MA	63.4%	10.6%	25.5%	0.5%
MU	100%	-	-	-
NA	61.4%	-	26.6%	12.1%
PV	65.9%	25.3%	6.7%	2.1%
VA	75.9%	-	21.6%	2.5%
SP	68.8%	11.9%	14.9%	4.5%

**Table 3:** Different possibilities of food loss and waste (FLW) management combining the waste hierarchy framework with regulatory issues limiting the use of animal products [40,42] and technical issues allowing the industrial use of recycled vegetable oils [43]. The check symbols and the cross marks highlights whether or not they can be managed in each option described. Background color highlight the management options considered in this work.

Food Loss and Waste Management	Cereals	Roots and Tubers	Sweets	Vegetable Oils	Vegetables	Fruits	Pulses	Meat	Fish and Seafood	Dairy Products	Eggs
Prevention	✔	✔	✔	✔	✔	✔	✔	✔	✔	✔	✔
Re-use	✔	✔	✔	✔	✔	✔	✔	🗶 ^a^	🗶 ^a^	🗶 ^a^	🗶 ^a^
Animal feed	✔	✔	✔	✔	✔	✔	✔	🗶 ^a^	🗶 ^a^	🗶 ^a^	🗶 ^a^
Industrial use	🗶	🗶	🗶	✔ ^b^	🗶	🗶	🗶	🗶	🗶	🗶	🗶
AC ^c^	✔	✔	✔	✔	✔	✔	✔	🗶 ^a^	🗶 ^a^	🗶 ^a^	🗶 ^a^
AD ^c^	✔	✔	✔	✔	✔	✔	✔	✔	✔	✔	✔
Thermal treatment ^c^	✔	✔	✔	✔	✔	✔	✔	✔	✔	✔	✔
Landfill ^c^	✔	✔	✔	✔	✔	✔	✔	✔	✔	✔	✔

^a^ regulatory issues, ^b^ technical issues, ^c^ FLW management considered in this work.

**Table 4:** Environmental impact categories assessed using the v.306 methodology of the Institute of Environmental Sciences of Leiden University.

Impact Category Group	Name	Acronym	Unit
Acidification	Acidification Potential	AP	kg SO_2_ Equivalent
Climate change	Global Warming Potential (excluding biogenic carbon) over 100 years	GWP	kg CO_2_ Equivalent
Depletion of abiotic resources	Abiotic Depletion Potential	ADP	kg Sb Equivalent
Eutrophication	Eutrophication Potential	EP	kg Phosphate Equivalent
Human Toxicity	Human Toxicity Potential	HTP	kg DCB Equivalent
Photochemical oxidation	Photochemical Ozone Creation Potential	POCP	kg Ethene Equivalent

## Appendix A

**Table A1:** Food loss and waste generation at each region (in tons), divided into the 11 food categories and four stages considered. Stages: 1: agricultural production, 2: processing and packaging, 3: distribution, and 4: consumption. Regions: AN: Andalusia, AR: Aragon, AS: Principality of Asturias, BA: Balearic Islands, CN: Canary Islands, CT: Cantabria, CM: Castile-La Mancha, CL: Castile and Leon, CAT: Catalonia, EX: Extremadura, GA: Galicia, LR: La Rioja, MA: Community of Madrid, MU: Region of Murcia, NA: Chartered Community of Navarra, PV: Basque Country, and VA: Valencian Community.

Spanish Region	Stage	Cereals	Roots and Tubers	Sweets	Vegetable Oils	Vegetables	Fruits	Pulses	Meat	Fish and Seafood	Dairy Products	Eggs
AN	1	258.6	55.1	5.1	21.8	362.0	360.4	10.2	20.6	13.5	33.0	7.4
	2	127.5	36.4	5.5	13.8	10.8	9.8	1.7	52.5	10.5	1.8	1.3
	3	21.1	16.0	7.0	3.2	59.1	48.6	3.8	39.6	11.1	5.8	6.3
	4	257.8	49.4	51.0	13.2	176.9	152.0	10.8	104.7	20.5	79.4	24.3
AR	1	140.9	30.0	1.4	11.9	197.2	196.4	5.6	27.1	0.2	24.0	14.2
	2	34.1	9.7	1.5	3.7	2.9	2.6	0.5	14.1	2.8	0.5	0.3
	3	3.3	2.5	1.1	0.5	9.3	7.6	0.6	6.2	1.8	0.9	1.0
	4	40.4	7.7	8.0	2.1	27.8	23.9	1.7	16.4	3.2	12.5	3.8
AS	1	2.2	2.3	0.0	0.0	24.2	28.4	0.9	0.0	0.0	0.0	0.2
	2	15.3	4.4	0.7	1.6	1.3	1.2	0.2	6.3	1.3	0.2	0.2
	3	2.6	2.0	0.9	0.4	7.4	6.1	0.5	5.0	1.4	0.7	0.8
	4	32.3	6.2	6.4	1.6	22.1	19.0	1.4	13.1	2.6	10.0	3.0
BA	1	14.7	3.1	0.2	1.2	20.6	20.5	0.6	1.7	2.9	3.4	0.6
	2	4.5	1.3	0.2	0.5	0.4	0.3	0.1	1.9	0.4	0.1	0.0
	3	2.8	2.1	1.0	0.4	7.8	6.4	0.5	5.2	1.5	0.8	0.8
	4	33.9	6.5	6.7	1.7	23.3	20.0	1.4	13.8	2.7	10.4	3.2
CN	1	22.0	4.7	0.4	1.9	30.8	30.6	0.9	0.6	6.5	1.0	3.7
	2	10.8	3.1	0.5	1.2	0.9	0.8	0.1	4.4	0.9	0.2	0.1
	3	5.3	4.0	1.8	0.8	14.8	12.2	0.9	9.9	2.8	1.4	1.6
	4	64.5	12.4	12.7	3.3	44.2	38.0	2.7	26.2	5.1	19.9	6.1
CT	1	15.7	3.3	0.4	1.3	22.0	21.9	0.6	3.3	1.2	7.2	0.2
	2	10.8	3.1	0.5	1.2	0.9	0.8	0.1	4.4	0.9	0.2	0.1
	3	1.5	1.1	0.5	0.2	4.1	3.4	0.3	2.8	0.8	0.4	0.4
	4	18.0	3.4	3.6	0.9	12.3	10.6	0.8	7.3	1.4	5.5	1.7
CM	1	234.6	50.0	2.5	19.8	328.4	327.0	9.3	18.2	4.2	32.0	34.3
	2	62.0	17.7	2.7	6.7	5.2	4.8	0.8	25.5	5.1	0.9	0.6
	3	5.2	3.9	1.7	0.8	14.5	11.9	0.9	9.7	2.7	1.4	1.5
	4	63.2	12.1	12.5	3.2	43.4	37.3	2.6	25.7	5.0	19.5	6.0
CL	1	278.2	59.2	3.6	23.5	389.4	387.7	11.0	35.8	0.6	59.8	23.2
	2	89.8	25.6	3.9	9.7	7.6	6.9	1.2	37.0	7.4	1.3	0.9
	3	6.2	4.7	2.1	1.0	17.4	14.3	1.1	11.7	3.4	1.7	1.9
	4	75.9	14.5	15.0	3.9	52.1	44.7	3.2	30.8	6.0	23.4	7.2
CAT	1	94.8	20.2	8.2	8.0	132.7	132.1	3.7	26.8	9.8	19.3	10.7
	2	204.7	58.4	8.9	22.1	17.3	15.7	2.7	84.4	16.9	3.0	2.1
	3	18.8	14.3	6.3	2.9	52.8	43.5	3.4	35.4	10.0	5.2	5.6
	4	230.4	44.1	45.6	11.8	158.1	135.9	9.7	93.6	18.4	71.0	21.7
EX	1	122.9	26.2	0.9	10.4	172.1	171.3	4.9	26.4	0.4	50.9	3.6
	2	20.7	5.9	0.9	2.2	1.7	1.6	0.3	8.5	1.7	0.3	0.2
	3	2.7	2.1	6.6	0.4	7.7	6.3	0.5	5.2	1.5	0.8	0.8
	4	33.5	6.4	2.8	1.7	23.0	19.8	1.4	13.6	2.7	10.3	3.2
GA	1	87.3	18.6	2.8	7.4	122.2	121.7	3.4	13.5	83.7	24.2	6.8
	2	69.1	19.7	3.0	7.5	5.9	5.3	0.9	28.5	5.7	1.0	0.7
	3	6.9	5.2	2.3	1.1	19.2	15.8	1.2	12.9	3.6	1.9	2.1
	4	83.9	16.1	16.6	4.3	57.5	49.5	3.5	34.0	6.7	25.8	7.9
LR	1	14.9	3.2	0.6	1.3	30.8	28.4	0.8	4.7	0.1	7.5	2.3
	2	15.3	4.4	0.7	1.6	1.3	1.2	0.2	6.2	1.3	0.2	0.2
	3	1.6	1.2	0.5	0.2	4.5	3.7	0.3	3.0	0.9	0.4	0.5
	4	1.6	1.2	0.5	0.2	4.5	3.7	0.3	3.0	0.9	0.4	0.5
MA	1	23.7	5.0	1.7	2.0	33.2	33.0	0.9	1.5	0.2	3.1	2.0
	2	43.1	12.3	1.9	4.7	3.6	3.3	0.6	17.8	3.6	0.6	0.4
	3	16.2	12.3	5.4	2.5	45.3	37.3	2.9	30.4	8.6	4.4	4.8
	4	197.6	37.8	39.1	10.1	135.6	116.5	8.3	80.2	15.7	60.9	18.6
MU	1	33.4	7.1	2.2	2.8	46.8	46.6	1.3	7.3	1.7	6.9	3.0
	2	54.8	15.6	2.4	5.9	4.6	4.2	0.7	22.6	4.5	0.8	0.5
	3	3.7	2.8	1.2	0.6	10.3	8.5	0.7	6.9	2.0	1.0	1.1
	4	45.0	8.6	8.9	2.3	30.9	26.6	1.9	18.3	3.6	13.9	4.2
NA	1	30.7	6.5	1.0	2.6	37.1	38.2	1.1	2.6	1.5	5.6	3.3
	2	26.0	7.4	1.1	2.8	2.2	2.0	0.3	10.7	2.1	0.4	0.3
	3	1.6	1.2	0.5	0.2	4.5	3.7	0.3	3.0	0.9	0.4	0.5
	4	19.7	12.9	1.9	0.1	1.8	0.0	0.0	0.0	0.0	0.0	0.0
PV	1	21.4	4.5	1.3	1.8	29.9	29.8	0.8	2.6	1.5	5.6	3.4
	2	32.3	9.2	1.4	3.5	2.7	2.5	0.4	13.3	2.7	0.5	0.3
	3	5.5	4.2	1.8	0.8	15.4	12.7	1.0	10.3	2.9	1.5	1.6
	4	67.2	12.9	13.3	3.4	46.1	39.6	2.8	27.3	5.4	20.7	6.3
VA	1	68.7	14.6	3.1	5.8	96.1	95.7	2.7	4.4	5.4	4.0	10.0
	2	77.2	22.0	3.4	8.3	6.5	5.9	1.0	31.8	6.4	1.1	0.8
	3	12.5	9.5	4.2	1.9	35.0	28.8	2.2	23.5	6.6	3.4	3.7
	4	152.9	29.3	30.2	7.8	104.9	90.2	6.4	62.1	12.2	47.1	14.4

**Table A2:** Relative variation (%) of impacts as compared to the current scenario (S1) per region for the considered S2 management scenario.

Scenario	Region	Framework	GWP	EP	AP	POCP	HT	ADP
S2	AN	BAU	26.4	−65.6	−62.2	−76.4	−90.4	−97.2
		2DS	18.9	−66.1	−63.6	−76.6	−90.4	−97.2
	AR	BAU	15.0	−19.3	−20.2	−11.6	−51.0	−17.3
		2DS	10.8	−19.6	−21.0	−11.7	−51.0	−17.3
	AS	BAU	1.4	−6.7	−6.0	−9.9	−4.6	−12.1
		2DS	1.1	−6.7	−6.1	−9.9	−4.6	−12.1
	BA	BAU	1.6	−6.4	−5.8	−8.9	−5.5	−11.0
		2DS	1.2	−6.4	−5.9	−8.9	−5.5	−11.0
	CN	BAU	2.5	−11.8	−10.6	−17.4	−8.1	−21.3
		2DS	1.8	−11.8	−10.7	−17.4	−8.1	−21.3
	CT	BAU	1.8	−4.6	−4.4	0.0	−6.1	−7.0
		2DS	1.3	−4.7	−4.5	−5.6	−6.1	−7.0
	CM	BAU	24.6	−31.6	−33.0	−18.9	−83.3	−28.3
		2DS	17.6	−32.1	−34.3	−19.2	−83.4	−28.3
	CL	BAU	29.4	−39.0	−40.5	−24.6	−100.0	−36.2
		2DS	21.1	−39.5	−42.0	−24.9	−100.0	−36.2
	CAT	BAU	10.6	−55.1	−49.4	−81.8	−36.8	−100.0
		2DS	7.5	−55.3	−50.0	−81.9	−36.9	−100.0
	EX	BAU	13.8	−16.4	−17.4	−8.4	−46.4	−13.2
		2DS	9.9	−16.6	−18.1	−8.6	−46.4	−13.2
	GA	BAU	11.3	−25.3	−24.3	−27.7	−38.7	−35.6
		2DS	8.1	−25.5	−24.9	−27.8	−38.7	−35.6
	LR	BAU	2.2	−5.0	−4.9	−5.6	−7.4	−7.1
		2DS	1.6	−5.0	−4.9	−5.6	−7.4	−7.1
	MA	BAU	2.4	−33.2	−28.7	−55.4	−8.5	−66.6
		2DS	1.7	−33.2	−28.8	−55.4	−8.5	−66.6
	MU	BAU	3.8	−12.9	−11.9	−17.5	−12.5	−21.7
		2DS	2.7	−13.0	−12.1	−17.5	−12.5	−21.7
	NA	BAU	3.1	−5.7	−5.6	−5.6	−10.3	−7.4
		2DS	2.2	−5.8	−5.8	−5.6	−10.3	−7.4
	PV	BAU	2.4	−13.6	−12.1	−20.7	−8.1	−25.2
		2DS	1.7	−13.7	−12.3	−20.7	−8.1	−25.2
	VA	BAU	7.1	−32.6	−29.4	−47.2	−24.5	−57.9
		2DS	5.1	−32.7	−29.8	−47.2	−24.5	−57.9

**Table A3:** Relative variation (%) of impacts as compared to the current scenario (S1) per region for the considered S3 management scenario.

Scenario	Region	Framework	GWP	EP	AP	POCP	HT	ADP
S3	AN	BAU	19.2	−61.9	−71.4	−80.7	−24.0	−97.2
		2DS	−3.2	−63.4	−75.5	−81.6	−24.1	−97.2
	AR	BAU	11.0	−17.3	−25.4	−14.0	−13.5	−17.3
		2DS	−1.7	−18.1	−27.7	−14.5	−13.5	−17.3
	AS	BAU	1.1	−6.5	−6.5	−10.1	−1.2	−12.1
		2DS	0.0	−6.5	−6.7	−10.1	−1.2	−12.1
	BA	BAU	1.2	−6.2	−6.4	−9.2	−1.5	−11.0
		2DS	−0.2	−6.2	−6.6	−9.2	−1.5	−11.0
	CN	BAU	1.8	−11.4	−11.4	−17.8	−2.2	−21.3
		2DS	−0.2	−11.6	−11.8	−17.8	−2.2	−21.3
	CT	BAU	1.3	−4.4	−5.0	−5.9	−1.6	−7.0
		2DS	−0.2	−4.5	−5.3	−5.9	−1.6	−7.0
	CM	BAU	17.9	−28.3	−41.4	−23.0	−22.1	−28.3
		2DS	−2.8	−29.6	−45.3	−23.7	−22.1	−28.3
	CL	BAU	21.4	−35.0	−50.6	−29.4	−26.5	−36.2
		2DS	−3.4	−36.6	−55.2	−30.3	−26.5	−36.2
	CAT	BAU	7.6	−53.6	−53.1	−83.6	−9.9	−100.0
		2DS	−1.5	−54.2	−54.8	−83.9	−9.9	−100.0
	EX	BAU	10.1	−14.5	−22.1	−10.7	−12.3	−13.2
		2DS	−1.5	−15.3	−24.2	−11.1	−12.3	−13.2
	GA	BAU	8.2	−23.7	−28.2	−29.5	−10.3	−35.6
		2DS	−1.4	−24.3	−30.0	−29.9	−10.3	−35.6
	LR	BAU	1.6	−4.7	−5.5	−5.9	−2.0	−7.1
		2DS	−0.2	−4.8	−5.9	−6.0	−2.0	−7.1
	MA	BAU	1.7	−32.8	−29.6	−55.8	−2.4	−66.6
		2DS	−0.4	−33.0	−30.0	−55.9	−2.4	−66.6
	MU	BAU	2.8	−12.4	−13.2	−18.1	−3.3	−21.7
		2DS	−0.3	−12.6	−13.7	−18.2	−3.4	−21.7
	NA	BAU	2.3	−5.3	−6.6	−6.1	−2.7	−7.4
		2DS	−0.3	−5.5	−7.1	−6.2	−2.7	−7.4
	PV	BAU	1.8	−13.3	−13.0	−21.1	−2.2	−25.2
		2DS	−0.2	−13.4	−13.3	−21.2	−2.2	−25.2
	VA	BAU	−0.1	−31.6	−31.9	−48.3	−6.6	−57.9
		2DS	−0.9	−32.0	−33.0	−48.6	−6.6	−57.9

**Table A4:** Relative variation (%) of impacts as compared to the current scenario (S1) per region for the considered S4 management scenario.

Scenario	Region	Framework	GWP	EP	AP	POCP	HT	ADP
S4	AN	BAU	−11.6	−95.7	−87.5	−97.6	−5.9	−97.2
		2DS	−77.2	−100.0	−100.0	−100.0	−6.0	−97.2
	AR	BAU	−6.4	−36.4	−34.5	−23.5	−3.3	−17.3
		2DS	−43.5	−38.8	−41.5	−24.9	−3.3	−17.3
	AS	BAU	−0.5	−8.2	−7.3	−10.9	−0.3	−12.1
		2DS	−3.8	−8.4	−7.9	−11.1	−0.3	−12.1
	BA	BAU	−0.7	−8.2	−7.3	−10.2	−0.4	−11.0
		2DS	−4.7	−8.5	−8.1	−10.4	−0.4	−11.0
	CN	BAU	−0.9	−14.5	−12.8	−19.3	−0.6	−21.3
		2DS	−6.8	−14.8	−14.0	−19.5	−0.6	−21.3
	CT	BAU	−0.7	−6.7	−6.1	−7.0	−0.4	−7.0
		2DS	−5.2	−7.0	−6.9	−7.2	−0.4	−7.0
	CM	BAU	−10.5	−59.4	−56.3	−38.5	−5.3	−28.2
		2DS	−71.1	−63.4	−67.9	−40.7	−5.4	−28.2
	CL	BAU	−12.7	−72.4	−68.5	−48.1	−6.4	−36.1
		2DS	−85.5	−77−2	−82.3	−50.8	−6.5	−36.1
	CAT	BAU	−4.9	−67.4	−59.7	−90.4	−2.5	−100.0
		2DS	−31.5	−69.1	−65.7	−91.4	−2.6	−100.0
	EX	BAU	−5.8	−31.9	−30.4	−19.3	−2.9	−13.2
		2DS	−39.5	−34.1	−36.8	−20.6	−3.0	−13.2
	GA	BAU	−5.0	−38.2	−35.1	−36.7	−2.5	−35.6
		2DS	−33.1	−40.0	−40.4	−37.8	−2.6	−35.6
	LR	BAU	−0.9	−7.5	−6.9	−7.3	−0.5	−7.1
		2DS	−6.3	−7.8	−7.9	−7.5	−0.5	−7.1
	MA	BAU	−1.2	−36.0	−31.1	−57.4	−0.7	−66.6
		2DS	−7.2	−36.4	−32.2	−57.6	−0.7	−66.6
	MU	BAU	−1.5	−17.1	−15.4	−20.4	−0.8	−21.7
		2DS	−10.6	−17.7	−17.1	−20.7	−0.9	−21.7
	NA	BAU	−1.2	−9.1	−8.5	−8.0	−0.7	−7.4
		2DS	−8.7	−9.6	−9.9	−8.3	−0.7	−7.4
	PV	BAU	−1.0	−16.3	−14.4	−22.6	−0.6	−25.2
		2DS	−6.9	−9.1	−15.5	−22.8	−0.6	−25.2
	VA	BAU	−3.2	−9.6	−36.3	−52.9	−1.7	−57.9
		2DS	−20.9	−16.3	−39.6	−53.6	−1.7	−57.9

**Table A5:** Relative variation (%) of impacts as compared to the current scenario (S1) per region for the considered S5 management scenario.

Scenario	Region	Framework	GWP	EP	AP	POCP	HT	ADP
S5	AN	BAU	34.6	−70.2	−74.2	−83.2	−40.7	−97.2
		2DS	1.8	−72.4	−80.4	−84.4	−40.8	−97.2
	AR	BAU	19.7	−22.0	−26.9	−15.4	−22.9	−17.3
		2DS	1.1	−23.2	−30.4	−16.1	−23.0	−17.3
	AS	BAU	1.9	−6.9	−6.6	−10.2	−2.1	−12.1
		2DS	0.2	−7.0	−6.9	−10.3	−2.1	−12.1
	BA	BAU	2.1	−6.7	−6.5	−9.3	−2.5	−11.0
		2DS	0.1	−6.8	−6.9	−9.4	−2.5	−11.0
	CN	BAU	3.2	−12.2	−11.6	−18.0	−3.7	−21.3
		2DS	0.3	−12.4	−12.2	−18.1	−3.7	−21.3
	CT	BAU	2.4	−5.0	−5.2	−6.0	−2.7	−7.0
		2DS	0.2	−5.1	−5.6	−6.1	−2.7	−7.0
	CM	BAU	32.1	−35.9	−44.0	−25.2	−37.5	−28.3
		2DS	1.8	−37.9	−49.7	−26.3	−37.5	−28.3
	CL	BAU	38.4	−44.2	−53.7	−32.1	−45.0	−36.2
		2DS	2.1	−46.5	−60.5	−33.5	−45.0	−36.2
	CAT	BAU	13.9	−57.0	−54.2	−84.6	−16.7	−100.0
		2DS	0.6	−57.9	−56.8	−85.1	−16.7	−100.0
	EX	BAU	18.0	−18.8	−23.5	−11.9	−20.8	−13.2
		2DS	1.1	−19.9	−26.7	−12.6	−20.9	−13.2
	GA	BAU	14.8	−27.2	−29.4	−30.6	−17.4	−35.6
		2DS	0.8	−28.2	−32.0	−31.1	−17.5	−35.6
	LR	BAU	2.8	−5.4	−5.8	−6.1	−3.3	−7.1
		2DS	0.2	−5.5	−6.3	−6.2	−3.4	−7.1
	MA	BAU	3.0	−33.6	−29.8	−56.0	−3.9	−66.6
		2DS	0.1	−33.8	−30.4	−56.2	−3.9	−66.6
	MU	BAU	4.9	−13.6	−13.6	−18.4	−5.7	−21.7
		2DS	0.4	−13.9	−14.4	−18.6	−5.7	−21.7
	NA	BAU	4.0	−6.2	−7.0	−6.4	−4.6	−7.4
		2DS	0.3	−6.5	−7.7	−6.5	−4.6	−7.4
	PV	BAU	3.1	−14.0	−13.2	−21.3	−3.7	−25.2
		2DS	0.2	−14.2	−13.8	−21.4	−3.7	−25.2
	VA	BAU	9.3	−33.8	−32.7	−49.0	−11.1	−57.9
		2DS	0.5	−34.4	−34.3	−49.3	−11.1	−57.9

**Table A6:** Relative variation (%) of impacts as compared to the current scenario (S1) per region for the considered S6 management scenario.

Scenario	Region	Framework	GWP	EP	AP	POCP	HT	ADP
S6	AN	BAU	45.1	−58.6	−66.3	−76.2	−60.1	−97.2
		2DS	31.5	−59.5	−68.8	−76.7	−60.1	−97.2
	AR	BAU	25.6	−15.4	−22.5	−11.4	−33.9	−17.3
		2DS	18.0	−15.9	−23.9	−11.7	−33.9	−17.3
	AS	BAU	2.4	−6.3	−6.2	−9.9	−3.1	−12.1
		2DS	1.7	−6.3	−6.3	−9.9	−3.1	−12.1
	BA	BAU	2.7	−5.9	−6.1	−8.9	−3.7	−11.0
		2DS	1.9	−6.0	−6.2	−8.9	−3.7	−11.0
	CN	BAU	4.1	−11.1	−10.9	−17.4	−5.4	−21.3
		2DS	2.9	−11.2	−11.2	−17.4	−5.4	−21.3
	CT	BAU	3.1	−4.2	−4.7	−5.5	−4.1	−7.0
		2DS	2.2	−4.2	−4.8	−5.6	−4.1	−7.0
	CM	BAU	41.8	−25.2	−36.7	−18.7	−55.4	−28.3
		2DS	29.3	−26.0	−39.0	−19.2	−55.4	−28.3
	CL	BAU	50.1	−31.3	−45.0	−24.3	−66.4	−36.2
		2DS	35.1	−32.2	−47.7	−24.9	−66.5	−36.2
	CAT	BAU	18.1	−52.3	−51.0	−81.7	−24.5	−100.0
		2DS	12.7	−52.6	−52.1	−81.9	−24.6	−100.0
	EX	BAU	23.4	−12.8	−19.4	−8.3	−30.8	−13.2
		2DS	16.4	−13.3	−20.7	−8.6	−30.8	−13.2
	GA	BAU	19.3	−22.3	−26.0	−27.6	−25.7	−35.6
		2DS	13.5	−22.6	−27.1	−27.8	−25.7	−35.6
	LR	BAU	3.7	−4.4	−5.1	−5.5	−4.9	−7.1
		2DS	2.6	−4.5	−5.3	−5.6	−4.9	−7.1
	MA	BAU	4.1	−32.5	−29.1	−55.4	−5.7	−66.6
		2DS	2.8	−32.6	−29.3	−55.4	−5.7	−66.6
	MU	BAU	6.4	−12.0	−12.5	−17.4	−8.3	−21.7
		2DS	4.5	−12.1	−12.8	−17.5	−8.4	−21.7
	NA	BAU	5.2	−4.9	−6.1	−5.6	−6.8	−7.4
		2DS	3.7	−5.0	−6.4	−5.6	−6.8	−7.4
	PV	BAU	4.1	−13.0	−12.5	−20.7	−5.4	−25.2
		2DS	2.9	−13.1	−12.7	−20.7	−5.4	−25.2
	VA	BAU	−0.2	−30.7	−30.5	−47.1	−16.3	−57.9
		2DS	8.5	−30.9	−31.2	−47.2	−16.3	−57.9
